# Oral Health and Hygiene Content in Nursing Fundamentals Textbooks

**DOI:** 10.1155/2012/372617

**Published:** 2012-04-09

**Authors:** Rita A. Jablonski

**Affiliations:** School of Nursing, The Pennsylvania State University, 201 Health and Human Development East, University Park, PA 16802, USA

## Abstract

The purpose of this paper is to describe the quantity and quality of oral hygiene content in a representative sample of before-licensure nursing fundamentals textbooks. Seven textbooks were examined. Quantity was operationalized as the actual page count and percentage of content devoted to oral health and hygiene. Quality of content was operationalized as congruency with best mouth care practices. Best mouth care practices included evidence-based and consensus-based practices as published primarily by the American Dental Association and supported by both published nursing research and review articles specific to mouth care and published dental research and review articles specific to mouth care. Content devoted to oral health and hygiene averaged 0.6%. Although the quality of the content was highly variable, nearly every textbook contained some erroneous or outdated information. The most common areas for inaccuracy included the use of foam sponges for mouth care in dentate persons instead of soft toothbrushes and improper denture removal.

## 1. Introduction

 Oral hygiene is vitally important because oral health is directly related to systemic health [1–3]. Poor oral health results in plaque buildup and inflammation of the gingiva. Plaque harbors pathogens associated with pneumonia [4]. In fact, poor oral hygiene has been linked to ventilator-associated pneumonia across the lifespan [5, 6]. Inflammation of the gingival tissues, either with or without periodontal disease, has been related to adverse outcomes in pregnancy, such as premature-birth and low-birth-weight infants [7]. Other systemic diseases associated with inadequate oral hygiene and resulting poor oral health include diabetes [8–10] and coronary artery disease [11]. Inadequate oral health negatively impacts quality of life and mortality, as well [12].

In 1986, Jones et al. surveyed nursing schools in the New England region to determine the quantity of oral health in both undergraduate and graduate curricula [13]. At the undergraduate level, Jones et al. reported an hour or less of overall oral health content in the entire curricula for 50% of the surveyed schools [13]. Fourteen percent of the undergraduate programs included 2 to 3 hours of oral health content specific to older adults; the remaining schools reported zero to 1 hour [13]. More recent reports of oral health content in undergraduate/predoctoral nursing, medical, and pharmacy schools show little, if any, improvement. Nearly 60 percent of educators in nursing, medicine, and pharmacology in English-speaking universities around the world currently describe their curricula in oral health as insufficient [14].

In 2009, at the request of the Department of Health and Human Services (DHHS), the Institute of Medicine convened an oral health panel. The panel, The Committee on an Oral Health Initiative, was charged with “assessing the current oral health care system, reviewing the elements of an HHS Oral Health Initiative, and exploring ways to promote the use of preventive oral health interventions and improve oral health literacy [15, page vii]. Members of the committee invited experts to share their experiences and perspectives during public meetings held across the United States. One area that members of the committee explored was the important contributions nondental clinicians make to the prevention, diagnosis, and treatment of oral diseases [15].The committee-desired information regarding the quantity and quality of oral health content in nursing education because nurses are responsible for either providing oral hygiene for their patients or supervising and delegating this task to unlicensed personnel [16]. This author was invited to address the committee and discuss the quantity and quality of oral health content in nursing education. In order to substantiate the content of the presentation, a search of nursing fundamentals textbooks was conducted in order to describe both the quantity and quality of oral hygiene content. Thus, the purpose of this paper is to describe the quantity and quality of oral hygiene content in before-licensure nursing fundamentals textbooks.

## 2. Materials and Methods

### 2.1. Search Description

 The purpose of this search was to obtain a representative sample of nursing fundamental textbooks in order to describe the quantity and quality of oral hygiene content. The Google search engine was used to conduct the textbook search because it interfaced with content found in the Google Book Projects. In 2007, Google and the Committee on Institutional Cooperation (CIC), a consortium of 12 universities, entered into a partnership that would allow Google to convert the millions of books owned by CIC libraries to electronic formats [17]. The goal was to digitize 10 million volumes. Many textbooks were not fully digitized due to copyright restraints, but the titles, table of contents, and other information are available for searching [17]. The search terms “nursing,” “fundamentals,” and “textbook” were used in the search. Only textbooks in English published from 2006 through 2010 were included; when multiple editions were identified, only the most recent edition was included in the sample. The intended audience for the textbooks was before-licensure registered nursing students; textbooks for before-licensure practical nursing students or nursing assistants were excluded. Study guides or companion books to the primary textbooks were excluded. Additionally, the same search terms and criteria were used to search Amazon and Barnes & Noble websites. No additional textbooks were identified. Finally, if a publisher of any nursing textbooks did not appear in these searches (such as SAGE), the website was searched as well. Seven fundamental textbooks were identified using these criteria.

 The textbooks were obtained via interlibrary loan. Quantity was operationalized as the actual page count and percentage of content devoted to oral health and hygiene in order to determine the quantity of oral hygiene content. Percentages were obtained by dividing the actual page count by the total pages of content and multiplying by 100. Total page count was determined by the last page of actual content, excluding indices, appendices, glossaries, and bibliographies. Quality of content was operationalized as congruency with best mouth care practices. Best mouth care practices included evidence-based and consensus-based practices as published primarily by the American Dental Association [18, 19] and supported by both published nursing research and review articles specific to mouth care and published dental research and review articles specific to mouth care [5, 6, 16, 20–28]. For example, nurse researchers have demonstrated the efficacy of specific oral health protocols, such as the use of soft toothbrushes instead of foam swabs for both dentate and edentate persons [27, 29–32]. Chalmers et al. [22] published a comprehensive evidence-based protocol for oral hygiene care targeting older adults with functional and cognitive impairments. Thus, contents in the nursing fundamental textbooks were examined for content congruent with oral hygiene practices tested and endorsed by nurses, dental hygienists, and dentists.

## 3. Results and Discussion

 Seven textbooks meeting the search criteria were obtained and are listed in Table 1. The percentage of oral health and hygiene content ranged from 0.27% [33] to 1.10% [34] with an average of 0.6%. Assessment of the oral cavity ranged from a few sentences [35] to 3.3 pages [34]. The assessment content in three textbooks [33, 35, 36] contained no information about assessing dentures for fit, integrity, or plaque. Potter and Perry [37] and Wilkinson and Van Leuven [34] offered the most complete information pertinent to oral health assessment. Potter and Perry [37] alone clearly articulated the oral-systemic link. This textbook also provided the clearest instructions for oral care with an unconscious or mechanically ventilated patient, for example, instructing the nurse to use an oral airway to keep the mouth of an unconscious or debilitated patient open. Three textbooks suggested using a tongue blade wrapped in gauze, which is not the safest or most comfortable approach [33, 35, 38]. Wilkinson and Van Leuven [34], on the other hand, recommended either the use of a tongue blade wrapped in gauze or a bite block.

 One textbook contained no information on how to correctly floss or brush teeth [36] such as brush at a 45 degree angle and use short strokes [19]. The same textbook, however, offered a recipe for toothpaste (2 parts baking soda, one part salt) without referencing the source of this information. The remaining six textbooks provided information on correct brushing techniques. On the other hand, content about flossing was problematic. The American Dental Association [19] recommends using 18 inches of string floss, winding the bulk of the floss around a finger of the nondominant hand, and using the dominant hand to spool the floss and take up the soiled sections as different teeth are flossed. While string floss is acceptable when assisting a cognitively intact patient with mouth care, floss holders and interdentate brushes are better choices when providing mouth care to dependent patients or those with cognitive impairments. In fact, the American Dental Association [19] does suggest floss holders and interdentate brushes for persons who have difficulty using string floss. Interdentate brushes, also called proximal brushes, resemble plastic toothpicks but with spiral shaped brushes on the end. These brushes are also perfect for nurses providing mouth care to fully dependent patients because the brushes allow the nurse to floss if the patient is unable or unwilling to open his or her mouth [40]. Furthermore, the use of interdentate brushes prevents bite injuries because the nurses' fingers are not in patients' mouths. In spite of these considerations, the authors of one textbook directed nurses to use string floss, instead of floss holders or interdentate brushes, when caring for a dependent patient [33]. The use of floss heads was recommended by Lynn [38] and Wilkinson and Van Leuven [34]. No textbooks contained recommendations for the use of interdentate brushes. Lynn [38] erroneously advised nurses to use 6 inches of string floss instead of the 18 inches as advised by the American Dental Association [18]. Flossing information provided by Delaune and Ladner [36] appeared contradictory. In one section of the textbook, nurses were advised to refrain from flossing the teeth of patients who were fully dependent on others for care. In another section, nurses were instructed to use floss holders when flossing the teeth of comatose patients.

Another problematic content area was the use of foam sponges in lieu of soft toothbrushes. Foam sponges do not remove plaque and debris as efficiently or completely as soft toothbrushes. Soft toothbrushes can be safely used for dentate patients, even unconscious ones [27]. In spite of the availability of this information since the mid-seventies [22], the use of foam sponges to provide oral hygiene was endorsed in some of the textbooks. Craven and Hirnle [35] and DeLaune and Ladner [36] advocated the use of a foam sponge to clean the teeth of dependent or unconscious patients. Wilkinson and Van Leuven [34] and Potter and Perry [37] explicitly stated that soft toothbrushes were superior to foam sponges but still recommended their usage.

 Regular toothpaste can contain particles that scratch acrylic denture material; the American Dental Association [18] recommends that regular toothpaste be avoided and suggests the use of household dish cleaning liquid for cleaning dentures. The authors of five textbooks promoted the use of toothpaste for denture cleaning [33, 34, 36, 38, 39]. Taylor et al. [33] provided no information on denture removal; the remaining six textbooks recommended the removal of top dentures first, followed by bottom dentures. In the dental and nursing literature, clinicians recommend removing the bottom denture first because it is easier to remove and minimizes bite risk for the caregiver [20, 22, 23]. Dentures also must be removed overnight to avoid damage to gingival surfaces and to prevent the growth of thrush on the hard palate. Yet, this important information was missing from two of the seven textbooks [34, 38].

 Only one textbook, Lynn [38], included content about oral hygiene and cognitively impaired older adults, but was vague regarding the best way to address care-resistant behavior. Given the aging of the American population [15, 41], registered nurses will find themselves caring for greater numbers of older adults and, very likely, older adults with cognitive impairments. All seven of the reviewed textbooks directed nurses to refer any dental problems, such as broken and loose teeth or poorly fitting dentures, to a dental professional.

This review was an attempt to systematically describe the quantity and quality of oral hygiene content in a representative sample of before-licensure nursing fundamentals textbooks. A strength of the search strategy was the use of identical search terms within multiple sources, which should have resulted in a representative sample of nursing fundamentals textbooks meeting the inclusion criteria. On the other hand, there is no primary database from which to identify nursing fundamentals textbooks. In spite of searching in a methodical manner and replicating the search within several sources, there exists the possibility that textbooks meeting the inclusion criteria may have been overlooked. Another limitation of this paper was the use of textbook titles and descriptions, and not the actual textbooks, in order to determine if the textbooks met the inclusion criteria before being obtained via interlibrary loan. It is possible that textbooks meeting the inclusion criteria may have been inadvertently excluded if the available title and description did not fully convey the intended audience or content.

## 4. Conclusion and Recommendations

 In conclusion, the oral health and hygiene content in these seven nursing fundamental textbooks were highly variable in quantity and quality. One challenge faced, by nurse authors writing the chapters and by nurse educators evaluating the content in the textbooks, was the lack of evidence-based guidelines addressing oral health and hygiene. For example, the sole evidence-based guidelines regarding the care and maintenance of dentures became available in 2011 [42]. These guidelines, however, do not provide concrete directives for the length of time dentures should be daily removed “While existing studies provide conflicting results, it is not recommended that dentures should be worn continuously (24 hours per day) in an effort to reduce or minimize denture stomatitis” [42, page S3]. Given the difficulty of obtaining accurate oral health and hygiene information without systematically poring through the dental and nursing literature, nurse educators are encouraged to engage in partnerships with dental professionals, especially those teaching in dental hygiene programs. In one such partnership, for example, dental hygiene faculty and students provided expertise in oral health assessments, while the nursing faculty and students shared their geriatric expertise with the entire team [31]. Nurse educators are also encouraged to incorporate the use of current clinical practice guidelines if the available textbooks do not contain the appropriate oral health content. Finally, it is imperative for nurse researchers involved in oral health and hygiene activities to actively engage in the dissemination of accurate information through publication and presentations. One such venue is the Oral Health Nursing Education & Practice Initiative within the New York University's College of Nursing at the College of Dentistry. This initiative was launched in April, 2011, and one of its goals includes disseminating best oral care practices to nurse educators [43].

 Oral health and hygiene has been an area overlooked in overall nursing education, but the growing body of research linking poor oral health to systemic diseases merits the need for added emphasis on the provision of oral hygiene [1–3]. In clinical practice, registered nurses provide oral hygiene either directly or supervise the provision of oral hygiene by others. Registered nurses who were not taught best mouth care practices may be providing inadequate mouth care as well as inadvertently promoting poor mouth care by unlicensed care personnel, who are dependent upon the knowledge and direction of registered nurses. It is important, therefore, for registered nurses to use current clinical mouth care practice guidelines.

## Figures and Tables

**Table 1:** Summary of Results.

Bibliographic data	Pages devoted to oral health and hhygiene	Total Pages*	Percent of oral health and hygiene content	Significant findings
				(i) Assessment was 0.25 page.
				(ii) Recommended cleaning dentures with soft-bristled toothbrush “because hard-bristled brushes can produce grooves in dentures” (page 722). Although this sentence is congruent with the American Dental Association guidelines [18, 19], it could be misconstrued as advising against using denture brushes, which tend to have firmer bristles than soft toothbrushes.
Craven and Hirnle [35]	4.5	1408	0.32%	(iii) Directed nursing student to use string floss, not floss heads or interdentate sticks.
				(iv) Suggested the use of a padded tongue blade oral to keep the mouth of an unconscious patient open.
				(v) Recommended either a soft toothbrush or foam swabs to brush teeth.
				(vi) Denture removal incongruent with published dental research.

				(i) Oral health assessment content did not address checking dentures for fit, integrity, or plaque.
				(ii) Recommended the use of foam swabs for “clients with impaired physical mobility or who are unconscious (comatose)” (page 759).
				(iii) No information on how to correctly floss or brush teeth, although the text provided a recipe for toothpaste: 2 parts salt and 1 part baking soda, no citations for this recipe.
				(iv) Recommended brushing dentures with toothpaste.
Delaune and Ladner [36]	8.5 with pictures	1391	0.61%	(v) Did not direct nurses to brush the bums of edentate patients with soft toothbrushes.
				(vi) Simultaneously recommended the use of foam sponges with toothpaste or toothbrushes with toothpaste.
				(vii) Confusing content in the area of flossing. For generic mouth care, solely advised the use of string floss for flossing. For patients who were comatose, directed the use of floss holders BUT also recommended against flossing teeth for patients fully dependent on nurses for care.
				(viii) Denture removal incongruent with published dental research.

				(i) Assessment content focused on the oral cavity (10 lines) but did not address normal versus abnormal findings.
				(ii) Included content specific to oral hygiene and persons with cognitive impairments consistent with published articles in this area by Chalmers [20–23].
				(iii) No mention of soft toothbrush, simply “toothbrush.”
				(iv) For assisting the patient, did recommend flossing, but technique was incorrect—recommended that the nurse use 6 inches of floss. Did recommend “a plastic floss holder” (page 338).
Lynn [38]	10	1042	0.96%	(v) Mouthwash is presented a simply a mechanism for “leaving a pleasant taste in the mouth” (page 338) instead as an adjuvant to prevent caries and gingivitis.
				(vi) Recommended use of a padded tongue blade to prop open the mouth of a “dependent” patient (page 341).
				(vii) For a dependent patient, recommended use of toothpaste and toothbrush.
				(viii) For a dependent patient, Remove dentures if present and use a foam swab or gauze-padded tongue blade “moistened with water or dilute mouthwash to gently clean gums, mucous membranes, and tongue.” (page 341).
				(ix) Denture removal incongruent with published dental research.
				(x) No direction about need to remove dentures overnight.
				(xi) Recommended toothpaste to brush dentures.

				
				(i) Out of all 7 textbooks, best description of oral health assessment (defined and described caries, periodontal disease, gingivitis; also addressed the components of an oral health assessment such as presence/absence of plaque quality of saliva and integrity of buccal mucosa).
				(ii) Oral-systemic link clearly articulated.
				(iii) Recommended brushing 4x/day.
				(iv) Stated that foam swabs) are ineffective and should not be used—but then, in procedure section, recommended foam swabs for unconscious or debilitated patients (page 889) and showed them in pictures.
Potter and Perry [37]	7.5	1408	0.53%	(v) Provided clearest instructions for oral care on an unconscious/mechanically ventilated patient.
				(vi) Recommended oral airway to keep mouth open for unconscious/debilitated patient.
				(vii) Foam swabs recommended for patients without teeth.
				(viii) Best description of oral health assessment, defined and described caries, periodontal disease, gingivitis.
				(ix) No mention of using toothpaste to clean dentures, but picture on page 891 shows toothpaste being used. (x) Denture removal and insertion directions simplistic too and incongruent with published dental research.
				(xi) No recommendation to use interdentate sticks or floss heads for flossing.

				(i) The oral health assessment was limited to 3 paragraphs in one health assessment chapter. There was no information on checking dentures for fit, integrity, or plaque. (ii) Provided overall correct mouth care techniques, including tongue brushing.
Taylor et al. [33]	4.75	1742	0.27%	(iii) Gave detailed directions for flossing using string floss; nothing about alternatives.
				(iv) For dependent patient, advised the use of padded tongue depressor to prop mouth open instead of a bite block.
				(v) Advised using toothpaste for dentures.
				(vi) No content on removing dentures from patient's mouth.
				(vii) Recommended mouth care every 1-2 hours, especially for persons who were not able to take anything by mouth.

				(i) Erroneously instructs patients in “Teaching Your Client About Oral Hygiene” to use regular toothpaste when brushing dentures.
Wilkinson and Leuven [39]	4.5	1089	0.41%	(ii) Also included a recipe for toothpaste, 1 part baking soda, 2 parts salt.
				(iii) Did not recommend removing and leaving dentures out overnight.

				(i) Included 3.3 pages about assessing the oral cavity; one of the most comprehensive.
				(ii) Included the use of foam swabs for mouth care, although the authors stated that toothbrushes better remove plaque and debris.
Wilkinson and Leuven [34]	11.3	1026	1.10%	(iii) Included content on the use of a floss holder.
				(iv) Recommended dilute hydrogen peroxide as a mouth wash.
				(v) Recommended toothpaste for cleaning dentures.
				(vi) Denture removal incongruent with published dental research.(vii) No indication for leaving dentures out overnight.
				(viii) For providing mouth care to an unconscious patient, recommended using a bite block or a padded tongue depressor.
